# Crystal structures and mechanical properties of osmium diboride at high pressure

**DOI:** 10.1038/s41598-021-85334-y

**Published:** 2021-03-11

**Authors:** Yi X. Wang, Ying Y. Liu, Zheng X. Yan, Wei Liu, Gao L. Zhou, Ke Z. Xiong

**Affiliations:** College of Science, Xi’an University of Science and Technology, Xi’an, 710054 People’s Republic of China

**Keywords:** Phase transitions and critical phenomena, Electronic structure

## Abstract

We have investigated the crystal structures and mechanical properties of osmium diboride (OsB_2_) based on the density functional theory. The structures of OsB_2_ from 0 to 400 GPa were predicted using the particle swarm optimization algorithm structure prediction technique. The orthorhombic *Pmmn* structure of OsB_2_ (*oP*6-OsB_2_) was found to be the most stable phase under zero pressure and it will transfer to the hexagonal *P*6_3_/*mmc* structure (*hP*6-OsB_2_) around 12.4 GPa. Meanwhile, we have discovered a new stable orthorhombic *Immm* structure (*oI*12-OsB_2_) above 379.6 GPa. After that, a thorough and comprehensive investigation on mechanical properties of different OsB_2_ phases is performed in this work. Further studies showed that the hardness of *oP*6-OsB_2_ and *hP*6-OsB_2_ at zero pressure is 15.6 and 20.1 GPa, while that for *oI*12-OsB_2_ under 400 GPa is 15.4 GPa, indicating that these three phases should be potentially hard materials rather than superhard materials. Finally, the pressure–temperature phase diagram of OsB_2_ is constructed for the first time by using the quasi-harmonic approximation method. Our results showed that the transition pressures of *oP*6-OsB_2_ → *hP*6-OsB_2_ and *hP*6-OsB_2_ → *oI*12-OsB_2_ all decreases appreciably with the increase of temperature.

## Introduction

Hard and superhard materials have attracted considerable attention because of their excellent performance as cutting tools and abrasives. Diamond is an excellent superhard material, but it is difficult to synthesize artificially^1,2^. Therefore, a lot of efforts have been invested to seek new hard materials in the past several decades. So far, one of the design principle of synthetic hard materials is to add light elements B, C, and N to electron-rich transition metals^3^, such as ReB_2_^4–7^, OsB_2_^8–12^, WB_4_^13,14^, PtC^15^, Re_2_C^16^, OsN_2_^17^, PtN_2_^18,19^, etc. Among them, the transition-metal borides have attracted considerable attention because of their excellent physicochemical property^20,21^.

OsB_2_ as a potential hard material, its structure and internal physical properties have been extensively studied in resent years^8–12,22–27^. To our best knowledge, OsB_2_ was first synthesized by Cumberland et al.^8^ through resistive heating method. They found that OsB_2_ possess the orthorhombic *Pmmn* structure (*oP*6-OsB_2_) at ambient condition, and it is an ultra-incompressible and hard material. It was soon confirmed by Gou et al.^22^ using first-principles total energy calculations, and showed that *oP*6-OsB_2_ is not a superhard material. Later, Gu et al.^9^ synthesized *oP*6-OsB_2_ compounds by arc melting and subsequent annealing method. They found that *oP*6-OsB_2_ does not belong to the superhard materials, and its stability can reach up to 34 GPa. Except the *oP*6-OsB_2_ phase, other structures of OsB_2_ have also been predicted by theoretical studies. For example, Hao et al.^28^ reported that the orthorhombic *Pnma* structure of OsB_2_ is thermodynamically and mechanically stable under environmental conditions. Moreover, using first-principles total energy calculations, Chen et al.^20^ predicted that two hexagonal structures of *P*6_3_/*mmc* and *P*6/*mmm* were also exist under pressure. Unfortunately, these phases have never been reported experimentally. Until 2014, Xie et al.^11^ successfully synthesized *hP*6-OsB_2_ phase by means of mechanochemical method, and it was found to be stable at ambient conditions. Then, Ren et al.^23^ investigated the structure stability of OsB_2_ under pressure through first-principles calculations. It is found that *oP*6-OsB_2_ is thermodynamically more stable than *hP*6-OsB_2_ at 0 GPa. With the increase of pressure, *oP*6-OsB_2_ will transfer to the *hP*6-OsB_2_ at 10.8 GPa. Most recently, by using particle swarm optimization algorithm, Feng et al.^27^ predicted two new high pressure phases *Fddd* and *Cmcm* of OsB_2_ under pressure of 0–100 GPa. But, it is worth noting that the two high-pressure phases were both metastable structures.

As mentioned above, previous studies^9,27,28^ have predicted several stable OsB_2_ structures under different pressures. However, the reported stable phases of OsB_2_ were not in agreement with each other. Furthermore, most of the studies on this problem mainly concentrated in the low pressure stage, and few studies have done on high pressures^23,27^. So far, the phase stability of OsB_2_ under pressure up to 400 GPa is still unknown. As a fundamental topic in condensed matter physics, revealing and elucidating the trend and mechanism of material high-pressure structural transformation is of great significance for its potential applications. Therefore, a thorough and comprehensive investigation on the phase stability of OsB_2_ under high pressure is really necessary. In this work, we explored the crystal structures of OsB_2_ from 0 to 400 GPa using particle swarm optimization (PSO) algorithm^29,30^ structure prediction technique. It is found that *oP*6-OsB_2_ is the most stable structure at 0 GPa and it will transition to *hP*6-OsB_2_ at low pressure range. Meanwhile, the orthorhombic *Immm* structure (*oI*12-OsB_2_) is predicted for the first time under high pressure, and it will be the most stable phase above 379.6 GPa. Furthermore, a comprehensive investigation on mechanical properties of different OsB_2_ phases also be performed in this work.

## Computational details

We performed the structure prediction of OsB_2_ at selected pressures of 0, 100, 200, 300, and 400 GPa using PSO algorithm^29^ as implemented in the CALYPSO code^30^. This method can predict stable or metastable crystal structures based on a given chemical compositions under specific external conditions. So far, it has been successfully applied not only to element solids, but also to binary and ternary compounds^31–34^. Moreover, the ab initio optimizations and mechanical properties calculations for every structure generated by the CALYPSO code were performed using the VASP package^35^ with the PBE generalized gradient approximation^36^. The electron–ion interactions was dealt with PAW pseudopotentials^37^ with 5*p*^6^5*d*^5^6*s*^2^ and 2*s*^2^2*p*^1^ valence configuration for Os and B, respectively. To achieve absolute convergences, the kinetic energy cutoff was set to 800 eV, and the Monkhorst–Pack *k*-point meshes^38^ was selected to 8 × 13 × 9 for *oP*6-OsB_2_, 16 × 16 × 6 for *hP*6-OsB_2_, 15 × 15 × 3 for *R-*3* m* structure (*hR*9-OsB_2_), 12 × 12 × 5 for *I*4*/mmm* structure (*tI*6-OsB_2_), 15 × 15 × 6 for *P*4/*nmm* structure (*tP*6-OsB_2_), 13 × 15 × 7 for *Cmcm* structure (*oC*12-OsB_2_), and 5 × 15 × 10 for *oI*12-OsB_2_. In addition, the phonon dispersion curves were calculated by using the finite displacement method^39^ within the PHON code^40^. To ensure that the force calculations were fully convergent, we used 2 × 4 × 3, 3 × 3 × 2, 3 × 3 × 3 supercells for *oP*6-OsB_2_, *hP*6-OsB_2_, *oI*12-OsB_2_ in the calculations. Meanwhile, the 3 × 4 × 3, 4 × 4 × 2, and 3 × 5 × 5 Γ-centered *k*-point meshes were chosen for *oP*6-OsB_2_, *hP*6-OsB_2_, and *oI*12-OsB_2_ supercells, respectively.

## Results and discussion

### Structure prediction and dynamical stability

**Figure 1:**
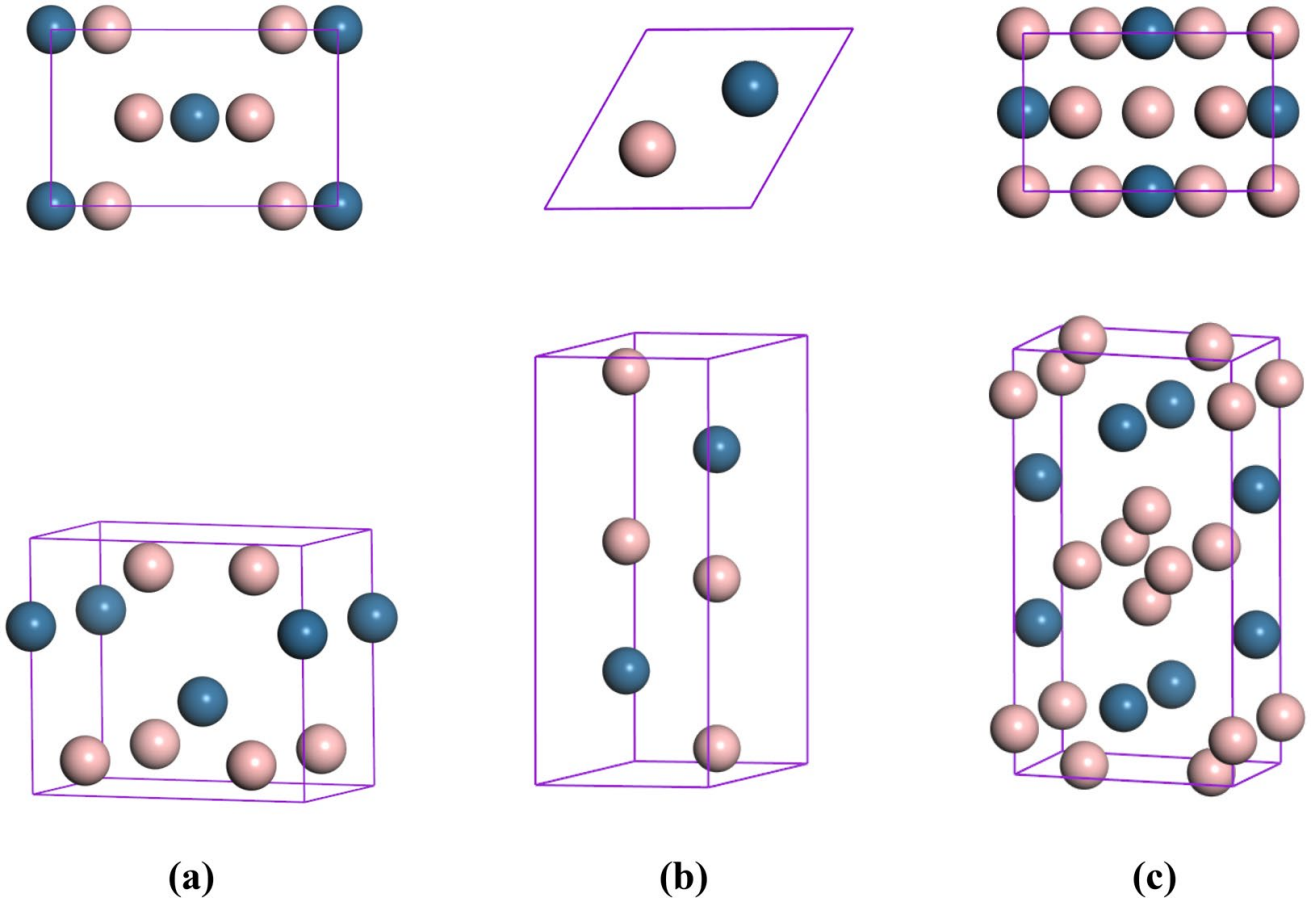
Top and three-dimensional views of the predicted crystal structures. (**a**) *oP*6-OsB_2_ at 0 GPa, (**b**) *hP*6-OsB_2_ at 20 GPa, and (**c**) *oI*12-OsB_2_ at 400 GPa.

According to the structure search results, *oP*6-OsB_2_, *hP*6-OsB_2_, *hR*9-OsB_2_, *tI*6-OsB_2_, *tP*6-OsB_2_, and *oC*12-OsB_2_ all possess lower enthalpy values under environmental pressure. Among them, *oP*6-OsB_2_ (Fig. 1a) is the most stable phase at 0 GPa, which is consistent with previous experimental^8,9,26^ and theoretical^22,23,27^ results. Moreover, we found that *hP*6-OsB_2_ (Fig. 1b) possesses the lowest enthalpy at the pressures of 100, 200 and 300 GPa. As the pressure increases further, a new *oI*12-OsB_2_ (Fig. 1c) structure is discovered for the first time, which is the most stable structure under 400 GPa. The predicted structural parameters of different OsB_2_ phases under pressure are listed in Table 1. For comparison the available experimental^9,26^ and theoretical^24,25,27^ data are also included. As shown, our obtained results agree well with other data, which proves the reliability of this research. In addition, we further evaluated the formation enthalpies of different OsB_2_ phases at ambient condition by the equation: ∆*H* = *H*(OsB_2_)—*H*(Os)—2*H*(B), in which the *R*-3* m* phase for B and the *Fm*-3* m* phase for Os were selected as the reference structures. Our obtained formation enthalpies of *oP*6-OsB_2_, *hP*6-OsB_2_, *hR*9-OsB_2_, *tI*6-OsB_2_, *tP*6-OsB_2_, *oC*12-OsB_2_, and *oI*12-OsB_2_ at zero pressure are -0.775, -0.751, -0.531, -0.476, -0.567, -0.633, and 0.910 eV/f.u., respectively. Among them, *oP*6-OsB_2_ possesses the minimum formation enthalpy. Moreover, except for the high-pressure phase *tI*12-OsB_2_, the formation enthalpies of other structures is all negative, indicating that they are potential metastable structures of OsB_2_ under environmental conditions.

**Table 1:** Lattice constants and atomic coordinates of different OsB_2_ structures, together with available experimental and theoretical results.

Space group	Pearson symbol	Parameters	Atom	x	y	z	Ref
Pmmn (0 GPa)	*oP*6	a = 4.702 b = 2.892 c = 4.092	Os(2a) B(4e)	0 0.3056	0 0.5	0.3449 0.1378	This work
		a = 4.6756 b = 2.8717 c = 4.0670	Os(2a) B(1a)	0 0.3049	0 0.5	0.3463 0.1383	Cal.^27^
		a = 4.696 c = 4.094					Cal.^24^
		a = 4.686 b = 2.876 c = 4.082					Exp.^9^
P63/mmc (0 GPa)	*hP*6	a = b = 2.916 c = 7.497	Os(2c) B(4f.)	0.3333 0.3333	0.6667 0.6667	0.25 0.5477	This work
		a = b = 2.9185 c = 7.3069	Os(2d) B(4f.)	0.3333 0.3333	0.6667 0.6667	0.25 0.4481	Cal.^27^
		a = b = 2.906 c = 7.301					Cal.^25^
		a = b = 2.922 c = 7.477					Exp.^26^
R-3 m (0 GPa)	*hR*9	a = b = 2.912 c = 11.191	Os(3a) B(6c)	0 0	0 0	0 0.1962	This work
		a = b = 2.890 c = 11.139	Os(3a) B(6c)	0 0	0 0	0 0.1965	Cal.^27^
I4/mmm (0 GPa)	*tI*6	a = b = 2.848 c = 6.840	Os(2a) B(4d)	0 0	0 0.5	0 0.25	This work
		a = b = 2.830 c = 6.803	Os(2b) B(4d)	0.5 0	0.5 0.5	0 0.25	Cal.^27^
P4/nmm (0 GPa)	*tP*6	a = b = 2.828 c = 6.751	Os(2c) B1(2c) B2(2b)	0 0 0.5	0.5 0.5 0.5	0.7539 0.833 0.5	This work
Cmcm (0 GPa)	*oC*12	a = 2.933 b = 7.247 c = 5.447	Os(4c) B(8f.)	0.5 0.5	0.9223 0.2226	0.75 0.5933	This work
Immm (400 GPa)	*oI*12	a = 2.418 b = 3.825 c = 7.172	Os(4j) B1(4i) B3(4 h)	0 0 0.5	0.5 0 0.2083	0.1703 0.1072 0	This work

**Figure 2:**
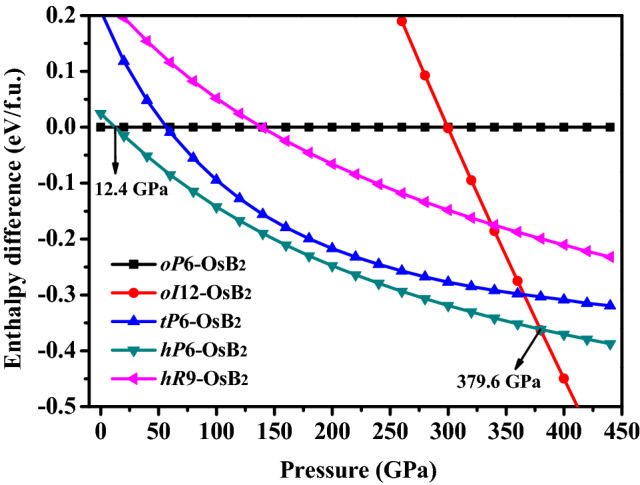
Enthalpy differences of predicted structures relative to *oP*6-OsB_2_ structure under high pressure.

**Figure 3:**
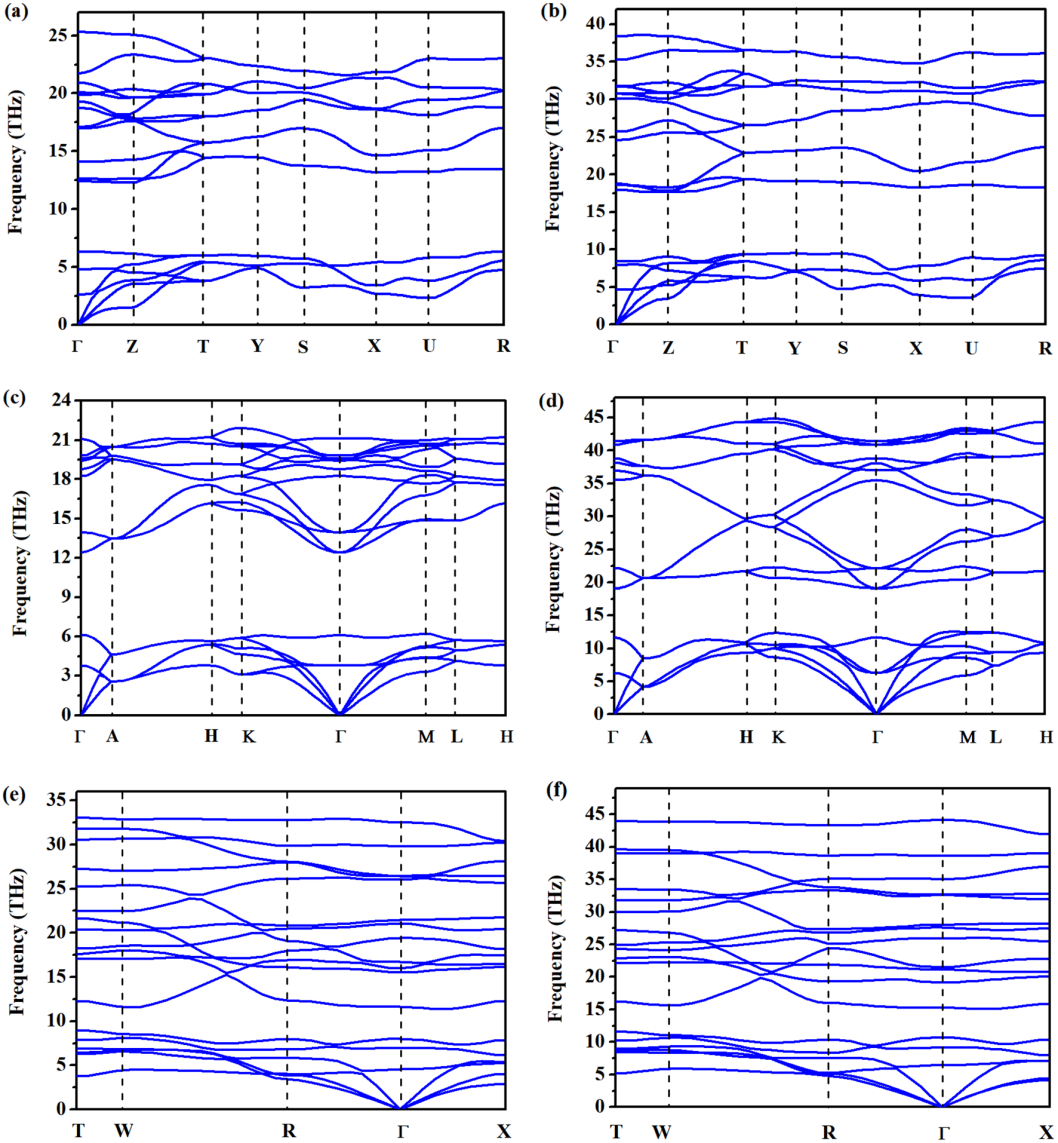
Calculated phonon dispersion curves of (**a**,**b**) *oP*6-OsB_2_ at 0 and 200 GPa, (**c**,**d**) *hP*6-OsB_2_ at 0 and 440 GPa, and (**e**,**f**) *oI*12-OsB_2_ at 200 and 440 GPa, respectively.

**Figure 4:**
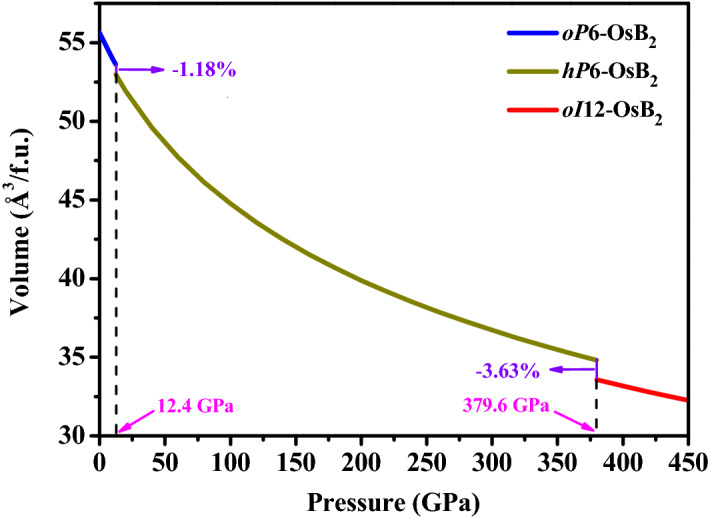
The equation of states (*P*–*V* curve) of OsB_2_. The purple lines indicate the volume reduction at 12.4 and 379.6 GPa.

To evaluate the transition pressures of OsB_2_ at zero Kelvin, the calculated enthalpy differences of *hP*6-OsB_2_, *hR*9-OsB_2_, *tP*6-OsB_2_, and *oI*12-OsB_2_ with respect to o*P*6-OsB_2_ phase under pressure are provided in Fig. 2. As shown, *oP*6-OsB_2_ has lower enthalpy than other structures at 0 GPa, indicating that it is the most stable structure under ambient conditions. Moreover, the pressure-induced transformation from *oP*6-OsB_2_ to *hP*6-OsB_2_ occurs at about 12.4 GPa, which is in good agreement with the theoretical result of 10.8 GPa^23^. After that, *hP*6-ReB_2_ possesses structure stability in a wide pressure range. As the pressure increases further, the stability of *oI*12-OsB_2_ gets enhanced gradually, and it becomes the most stable phase above 379.6 GPa. Thus, the phase transition sequence of OsB_2_ under pressure should be *oP*6-OsB_2_ → *hP*6-OsB_2_ → *oI*12-OsB_2_. Moreover, the vibrational phonon dispersion curves under a series of pressures are further calculated to assess their dynamic stability. The obtained results at selected pressures are presented in Fig. 3. We found that o*P*6-OsB_2_, *hP*6-OsB_2_, and *oI*12-OsB_2_ do not show any imaginary frequencies under pressure of 0–200, 0–440, and 200–440 GPa, respectively, indicating that these phases should be dynamically stable within the corresponding pressure range. In addition, the equations of state of OsB_2_ under pressure up to 450 GPa is further evaluated, as shown in Fig. 4. It is obvious that the volume decreases of 1.18% from o*P*6-OsB_2_ to *hP*6-OsB_2_ and 3.63% from *hP*6-OsB_2_ to *oI*12-OsB_2_, which means that the pressure-induced structure transitions of OsB_2_ are first-order rather than continuous.

### Mechanical properties

The elastic properties of OsB_2_ under different pressures are investigated by strain–stress method. Our calculated elastic constants of *oP*6-OsB_2_, *hP*6-OsB_2_, *hR*9-OsB_2_, *tI*6-OsB_2_, *tP*6-OsB_2_, and *oC*12-OsB_2_ under zero pressure are given in Table 2. It is obvious that our obtained results are consistent well with the available theoretical data^24,25,27,41^. Moreover, the obtained elastic constants of *oI*12-OsB_2_ at 400 GPa are also included in Table 2, but unfortunately there is no data can be compared with them. In addition, we can judge the mechanical stability of crystals based on their elastic constant^42,43^. For hexagonal structures of *hP*6-OsB_2_ and *hR*9-OsB_2_,

1 $$ \tilde{C}_{44} > 0,\tilde{C}_{11} - \left| {\tilde{C}_{12} } \right| > 0,\left( {\tilde{C}_{11} + \tilde{C}_{12} } \right)\tilde{C}_{33} - 2\tilde{C}_{13}^{2} > 0. $$

**Table 2:** Calculated elastic constants *C*_ij_ (in GPa) of different OsB_2_ structures, together with other theoretical results.

Phase	*C*_11_	*C*_12_	*C*_13_	*C*_22_	*C*_23_	*C*_33_	*C*_44_	*C*_55_	*C*_66_	Ref
*oP*6-OsB_2_ (0 GPa)	541	176	180	565	125	763	194	67	191	This work
	586	201	179	551	161	792	206	54	222	Cal.^27^
	549	164	183	538		744	203	77	199	Cal.^24^
	570	178	188	540		753	191	68	192	Cal.^41^
*hP*6-OsB_2_ (0 GPa)	478	179	216			887	212		150	This work
	404	178	294			873	227		113	Cal.^27^
	487	180	229			880	215		154	Cal.^25^
	453	183	218			870	206			Cal.^24^
*hR*9-OsB_2_ (0 GPa)	511	183	181			902	249		164	This work
	505	209	215			929	262		148	Cal.^27^
*tI*6-OsB_2_ (0 GPa)	627	66	194			913	301		153	This work
	650	80	214			969	306		107	Cal.^27^
*tP*6-OsB_2_ (0 GPa)	702	92	185			872	175		171	This work
*oC*12-OsB_2_ (0 GPa)	504	135	162	598	93	672	247	151	110	This work
*oI*12-OsB_2_ (400 GPa)	2619	1086	1397	2611	1313	2145	485	496	408	This work

For tetragonal crystals of *tI*6-OsB_2_ and *tP*6-OsB_2_,

$$ \tilde{C}_{ii} > 0,\quad \left( {i = \, 1, \, 3, \, 4, \, 6 \, } \right), $$

2 $$ \tilde{C}_{11} - \tilde{C}_{12} > 0,\tilde{C}_{11} + \tilde{C}_{33} - 2\tilde{C}_{13} > 0,2\left( {\tilde{C}_{11} + \tilde{C}_{12} } \right) + \tilde{C}_{33} + 4\tilde{C}_{13} > 0. $$

For orthorhombic phases of *oP*6-OsB_2_, *oC*12-OsB_2_ and *oI*12-OsB_2_,

$$ \tilde{C}_{ii} > 0,\left( {i = \, 1, \, 2, \, 3, \, 4, \, 5, \, 6 \, } \right), $$

$$ \tilde{C}_{11} + \tilde{C}_{22} + \tilde{C}_{33} + 2\left( {\tilde{C}_{12} + \tilde{C}_{13} + \tilde{C}_{23} } \right) > 0, $$

3 $$ \tilde{C}_{ii} + \tilde{C}_{jj} - 2\tilde{C}_{ij} > 0,\left( {i,j = \, 1, \, 2, \, 3,i \ne j} \right). $$

where $\tilde{C}_{ii} = C_{ii} - P$ (*i* = 1, 2, 3, 4, 5, 6), $\tilde{C}_{ij} = C_{ij}$ (*i* = 1, 2, 3; *j* = 4, 5, 6), $\tilde{C}_{12} = C_{12} + P,$ $\tilde{C}_{13} = C_{13} + P,$ $\tilde{C}_{23} = C_{23} + P,$ $\tilde{C}_{45} = C_{45} ,$ $\tilde{C}_{46} = C_{46} ,$ $\tilde{C}_{56} = C_{56} .$ After careful check, the calculated elastic constants of *oP*6-OsB_2_, *hP*6-OsB_2_, *hR*9-OsB_2_, *tI*6-OsB_2_, *tP*6-OsB_2_, *oC*12-OsB_2_, and *oI*12-OsB_2_ all meet their mechanical stability conditions, which means that these structures should be mechanically stable under the corresponding pressure.

In addition, for all OsB_2_ structures, the calculated *C*_11_, *C*_22_, and *C*_33_ are obviously larger than other elastic constants, indicating that they should be hard to compress on the *a*, *b*, and *c* axes. As the hardest natural minerals, diamond possesses a high elastic constant *C*_11_ of 1079 GPa at ambient conditions^44^. Interestingly, our obtained *C*_33_ of *hP*6-OsB_2_ is about 887 GPa at zero pressure, close to the *C*_11_ value of diamond, which implies that *hP*6-OsB_2_ may also be a hard material. For this reason, we further evaluated the hardness of different OsB_2_ structures by an empirical model^45^, which can be expressed as

4 $$ H_{v} = \, 2\left( {k^{2} G} \right)^{0.585} - 3, $$

in which *k* is equal to *G*/*B.* Through Voigt-Reuss-Hill approximation^46^, the bulk modulus *B*, and shear modulus *G* can be calculated by the elastic constants *C*_ij_. The obtained results, along with the other theoretical data^24,25,27,41^, are presented in Table 3. It is obvious that our calculated *B* and *G* agree with the available calculated results. Moreover, we can judge the ductility or brittleness of a material by the *B*/*G* ratio^47^. Usually, a material is ductile if *B*/*G* > 1.75, or else it is brittle. Under zero pressure, our calculated *B*/*G* of *oP*6-OsB_2_, *hP*6-OsB_2_, *hR*9-OsB_2_, *tI*6-OsB_2_, *tP*6-OsB_2_, and *oC*12-OsB_2_ is 1.90, 1.71, 1.50, 1.30, 1.62, and 1.54, respectively. This indicates that *oP*6-OsB_2_ is ductile, and the other phases are all brittle under environmental conditions. Meanwhile, the high-pressure phase *oI*12-OsB_2_ should be ductile at 400 GPa, because its *B*/*G* ratio is greater than the critical value under this pressure.

**Table 3:** Calculated bulk modulus *B* (in GPa), shear modulus *G* (in GPa), *B*/*G*, Young’s modulus *E* (in GPa), Poisson’s ratio *σ*, and hardness *H*_V_ (in GPa) of different OsB_2_ structures, together with available experimental and theoretical results.

	*B*	*G*	*B/G*	*E*	*σ*	*H*_V_	Ref
*oP*6-OsB_2_ (0 GPa)	312	164	1.90	419	0.28	15.6	This work
	335	189	1.77	477	0.263	19.6	Cal.^27^
	304	172	1.77	434	0.262	21.9	Cal.^24^
	310	164	1.89	419	0.275		Cal.^41^
				410	0.27	21.6	Exp.^4^
*hP*6-OsB_2_ (0 GPa)	326	191	1.71	480	0.25	20.1	This work
	334	192	1.74	485	0.258	34.5	Cal.^25^
	357	174	2.05	449	0.290	15.7	Cal.^27^
*hR*9-OsB_2_ (0 GPa)	325	217	1.50	533	0.23	26.0	This work
	357	221	1.62	550	0.243	24.4	Cal.^27^
*tI*6-OsB_2_ (0 GPa)	331	255	1.30	609	0.19	34.7	This work
	365	261	1.40	632	0.211	32.3	Cal.^27^
*tP*6-OsB_2_ (0 GPa)	351	217	1.62	540	0.24	23.5	This work
*oC*12-OsB_2_ (0 GPa)	283	184	1.54	453	0.23	22.5	This work
*oI*12-OsB_2_ (400 GPa)	1661	503	3.30	1372	0.36	15.9	This work

According to the obtained *B* and *G*, we can further calculated Young’s modulus *E* and Poisson’s ratio *σ* by

5 $$ E = \frac{9BG}{{3B + G}},\quad \sigma = \frac{3B - 2G}{{6B + 2G}}. $$

As shown in Table 3, our calculated results are consistent well with the available experimental^4^ or other calculated data^24,25,27,41^. Generally, a higher value of *E* indicates that the material is stiffer, while a smaller value of *σ* indicates that the covalent bond is more directional. Accordingly, the order of stiffness of OsB_2_ is *tI*6 > *tP*6 > *hR*9 > *hP*6 > *oC*12 > *oP*6, while the directionality degree of covalent bonds of OsB_2_ should be *tI*6 > *hR*9 > *oC*12 > *tP*6 > *hP*6 > *oP*6 under ambient conditions. Furthermore, our calculated hardness of *oP*6-OsB_2_, *hP*6-OsB_2_, *hR*9-OsB_2_, *tI*6-OsB_2_, *tP*6-OsB_2_, and *oC*12-OsB_2_ under zero pressure is 15.6, 20.1, 26.0, 34.7, 23.5, and 22.5 GPa, respectively, which agree well with the available experimental^4^ and theoretical^24,25,27,41^ results. Meanwhile, the high-pressure phase *oI*12-OsB_2_ possesses the hardness of 15.9 GPa at 400 GPa. Usually, the hardness of superhard materials should be higher than 40 GPa^48,49^. Accordingly, all the OsB_2_ phases studied above can be used as the candidate for hard materials rather than superhard materials.

### *Pressure*–*temperature phase diagram*

After established the phase transition sequence of *oP*6-OsB_2_** → hP**6-OsB_2_** → oI**12-OsB_2_ under pressure at zero Kelvin. We further investigated the stable region of each phases under high pressure and high temperature through the quasi-harmonic approximation (QHA)^50^ method. In which, the Helmholtz free energy is given by

6 $$ F\left( {V,T} \right) = E\left( V \right) + F_{{{\text{vib}}}} \left( {V,T} \right), $$

where *E*(*V*) is the static energy, *F*_vib_(*V*,*T*) is the nonequilibrium vibrational Helmholtz free energy

7 $$ F_{vib} \left( {V,T} \right) = \int_{0}^{\infty } {\left[ {\frac{\hbar \omega }{2} + \kappa_{B} T\ln \left( {1 - e^{{ - \hbar \omega /\kappa_{B} T}} } \right)} \right]} g\left( {\omega ,T} \right)d\omega , $$

in which *g*(*ω*,*V*) represents phonon density of state. Then, we can get the Gibbs free energy by

8 $$ G_{{{\text{gibbs}}}} = F\left( {V,T} \right) - V\left( {\frac{\partial F}{{\partial V}}} \right)_{T} . $$

**Figure 5:**
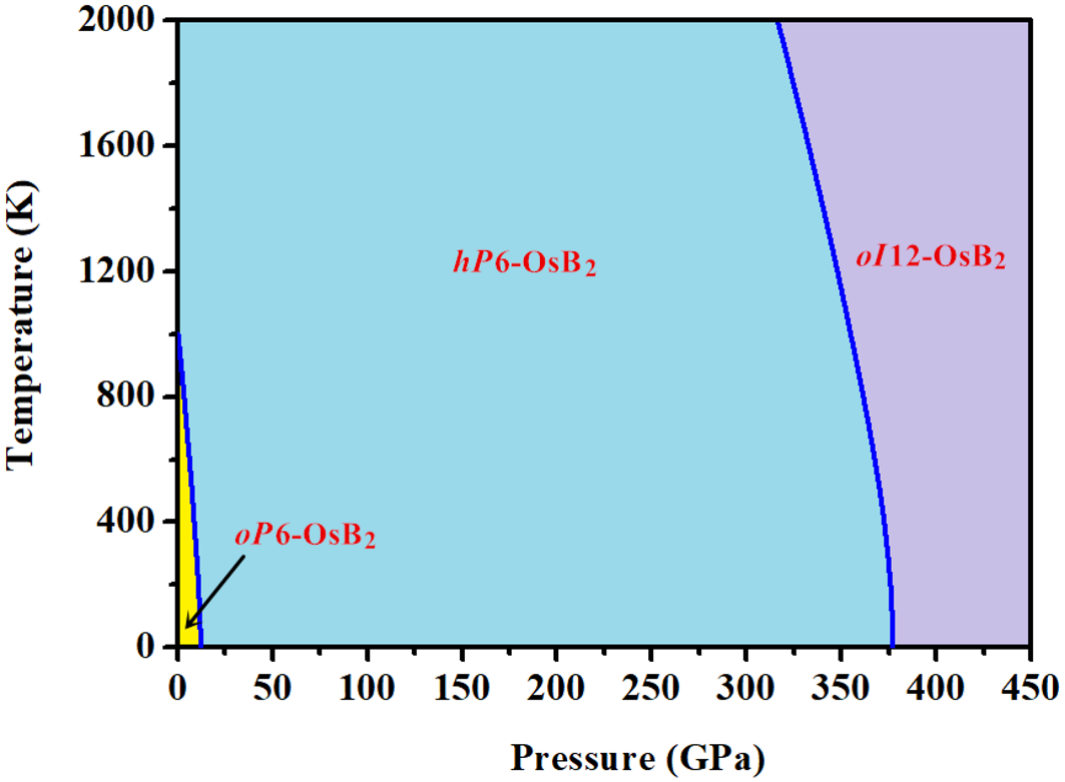
Calculated temperature–pressure phase diagram of OsB_2_ based on the QHA.

Our phonon frequencies calculations in Sect. 3.1 indicates that *oP*6-OsB_2_, *hP*6-OsB_2_, and *oI*12-OsB_2_ are dynamically stable within a large pressure range. Thus, according to the calculated Gibbs free energies of these three phases under different temperatures and pressures, the phase diagram of OsB_2_ is constructed for the first time, as shown in Fig. 5. It can be seen that temperature has a significant effect on the structural stability of OsB_2_. With the increase of temperature, the transition pressures of *oP*6-OsB_2_ → *hP*6-OsB_2_, and *hP*6-OsB_2_ → *oI*12-OsB_2_ all decreases appreciably. Moreover, *oP*6-OsB_2_ is located only in a very small region at the lower left corner of phase diagram, and it will transition to *hP*6-OsB_2_ with a temperature of ~ 1000 K at zero pressure. This is consistent with the previous experimental results of 873 K^12^. Meanwhile, the phase transformation of *hP*6-OsB_2_ → *oI*12-OsB_2_ occurs at 374.5 GPa under room temperature of 300 K.

## Conclusions

We have investigated the structure stability of OsB_2_ from 0 to 400 GPa through the ab initio PSO algorithm. The phase transition sequence of *oP*6-OsB_2_ → *hP*6-OsB_2_ → *oI*12-OsB_2_ under pressure was established. Through high precision calculations, the transition pressures were determined as 12.4 and 379.6 GPa at zero Kelvin. Phonon frequencies calculations show that *oP*6-OsB_2_, *hP*6-OsB_2_, and *oI*12-OsB_2_ possess dynamical stability under 0–200, 0–440, and 200–440 GPa, respectively. Moreover, the elastic constants and elastic-dependent properties of OsB_2_ are also successfully investigated. Our calculated hardness suggests that *oP*6-OsB_2_, *hP*6-OsB_2_, and *oI*12-OsB_2_ are both potential hard materials rather than superhard materials. Furthermore, the phase diagram of OsB_2_ at high pressure and high temperature was constructed for the first time based on the QHA method. Our result shows that the transition pressures of *oP*6-OsB_2_ → *hP*6-OsB_2_ and *hP*6-OsB_2_ → *oI*12-OsB_2_ all decreases with the increase of temperature.
